# Community perceptions and practices on hepatic veno-occlusive disease in Tigray, Ethiopia: An explorative study challenging the attribution to *Ageratum conyzoides*

**DOI:** 10.1371/journal.pntd.0013621

**Published:** 2025-10-15

**Authors:** Mekonnen Haileselassie, Yimer Mulugeta Agga, Ataklti Gebretsadik, Ziada Abdelhadi Abdelwahab, Fisseha Ashebir, Mulugeta Woldu Abrha, Brhane Ayele, Hailay Gebretnsae, Hagos Degefa Hidru, Hayelom Kahsay, Tsegay Hadgu, Yemane Berhane Tesfau, Abreha Tesfaye Genzebu, Feyisa Regessa, Geremew Tasew, Getachew Tollera, Mesay Hailu, Abiy Girmay Haddis, Senait Tekeste Fekadu, Patrick Okumu Abok, Samuel Aregay, Mebrahtu Hafte, Amanuel Haile, Mussie Alemayehu, Afework Mulugeta

**Affiliations:** 1 Tigray Health Research Institute, Mekelle, Tigray, Ethiopia; 2 Ethiopian Public Health Institute, Addis Ababa, Ethiopia; 3 Mekelle University, College of Business and Economics, Mekelle, Tigray, Ethiopia; 4 Adigrat University, College of Health Science, Adigrat, Tigray, Ethiopia; 5 Mekelle University, College of Veterinary Medicine, Mekelle, Tigray, Ethiopia; 6 World Health Organization (WHO), Addis Ababa, Ethiopia; 7 Tigray Regional Health Bureau, Mekelle, Tigray, Ethiopia; 8 Mekelle University, College of Health Sciences, School of Public Health, Mekelle, Tigray, Ethiopia; KARI-Trypanosomiasis Res Centre, KENYA

## Abstract

**Background:**

Hepatic veno-occlusive disease (HVOD) is a rare but severe condition characterized by the blockage of microscopic veins in the liver due to endothelial damage, leading to sub-endothelial thickening, edema, and fibrosis. Globally the cause of HVOD is primarily associated with pyrrolizidine alkaloid (PA) ingestion, radiation therapy, and post-transplant reactions. Similarly, in the Tigray region of Ethiopia, the disease’s outbreak has been linked with contaminated harvests containing PA-producing seed called “*Ageratum conyzoides”.* However, the perception and current experience of community members and healthcare providers on the cause and prevention strategies of the disease are not explored.

**Objective:**

To explore the perceptions and practices of community members and healthcare providers on HVOD in Tigray, Ethiopia.

**Methods:**

This qualitative study was conducted in the Tigray Region of Northern Ethiopia from January to February 2025. It employed a community and facility-based approach using in-depth interviews (IDIs), focus group discussions (FGDs), and key informant interviews (KIIs) to explore perceptions and current experiences on the causes and practice of preventive measures of HVOD. We selected five districts with high HVOD burdens. We included religious leaders and HVOD victims from the community members and healthcare leaders and professionals from the health facilities. A total of three FGDs, sixteen IDIs, and seven KIIs were conducted and data was analyzed thematically using Atlas.ti software.

**Results:**

The community’s understanding of HVOD is complex, recognizing it as a severe chronic disease but with uncertainty about its transmission and prevention. Our study identified three key themes: variability in knowledge among community members and healthcare providers regarding HVOD’s causes, prevention, and treatment, alongside a notable lack of coordinated leadership and support from healthcare providers, political leaders, and other stakeholders. Participants expressed frustration over the absence of structured interventions for awareness, prevention, and treatment. Additional barriers included skepticism about Ageratum conyzoides as the cause, social stigma, traditional beliefs, political instability, healthcare system weaknesses, and economic challenges. Acceptance of scientific interventions was uneven, shaped by varying levels of trust and resistance. Participants emphasized the need for targeted healthcare provider training, stronger community engagement in awareness and planning, and formal integration of HVOD into national health programs to improve resource allocation and coordination. These findings highlight the complex challenges and inform strategies for more effective HVOD control.

**Conclusions:**

Hepatic veno-occlusive disease (HVOD) is a significant public health issue in Ethiopia, which is exacerbated by misconceptions and systemic healthcare challenges. To address this, strengthening healthcare systems and community engagement through awareness campaigns are crucial. Integrating HVOD into the national public health emergency management programs with multi-sectoral collaboration could be essential for its effective management.

## Author summary

Hepatic veno-occlusive disease (HVOD) is characterized by the obstruction of small hepatic veins resulting from endothelial cell injury, leading to sub-endothelial thickening, edema, and fibrosis. This condition is frequently associated with the ingestion of pyrrolizidine alkaloids (PAs). In Tigray, Ethiopia, HVOD outbreaks are linked to crop contamination by *Ageratum conyzoides* seeds, which produce toxic pyrrolizidine alkaloids, yet community and healthcare provider perceptions of the disease and its prevention remain poorly understood. This qualitative study explored these perceptions through in-depth interviews, focus group discussions, and key informant interviews across five high-burden districts in Tigray. Results revealed complex community understanding, with recognition of HVOD’s severity but confusion about its transmission and prevention. Skepticism about the link to *Ageratum* complicates control efforts, while social stigma discourages early healthcare seeking. Healthcare providers face challenges including limited training, resource constraints, and the impact of ongoing conflict. Participants recommended enhanced healthcare training, community awareness campaigns, and multi-sectoral collaboration to strengthen surveillance and support. Addressing these barriers is critical for effective HVOD management and integrating the disease into national public health emergency programs could improve outcomes in affected populations.

## Introduction

Hepatic veno-occlusive disease (HVOD) involves blockage of liver veins due to endothelial cell damage, causing sub-endothelial thickening, edema, and fibrosis [1]. It has high mortality rates and substantial economic impact from both direct medical costs and indirect expenses [2,3]. It is caused by pyrrolizidine alkaloid ingestion, radiation, or post-transplant reactions, affecting all ages. Symptoms include hepatomegaly, jaundice, edema, and ascites, with severe cases leading to hepatic and renal insufficiency, organ failure, and infections [4–7].

In developed countries, HVOD is primarily linked to hematopoietic stem cell transplantation, affecting under 10% of patients [1]. It also occurs after chemotherapy or radiation. Hepatic veno-occlusive disease (HVOD) from pyrrolizidine alkaloids is uncertain but reported in Ethiopia, possibly due to contaminated harvests [8,9].

Pyrrolizidine alkaloids (PAs) are toxic plant metabolites produced as a defense mechanism against herbivores. These compounds are found in various foods, including spices, honey, and herbal teas, posing a health risk upon consumption due to their widespread presence in plant-derived products [10,11]. Approximately three percent of the world’s flowering plants contain one or more of these hazardous PAs. Hepatic veno-occlusive disease (HVOD) can be caused by consuming contaminated products, such as flour contaminated with wild weeds collected alongside grain crops [12]. The development of HVOD from PA consumption is dosage-dependent, meaning that not all exposure to PA-containing foods or plants will result in this condition [13].

Treatment for HVOD, primarily involves supportive care and specific drug therapy. Defibrotide is a common drug for treating HVOD, particularly after hematopoietic stem cell transplantation. It works by protecting endothelial cells and restoring the thrombotic-fibrinolytic balance. Supportive measures include careful fluid management with diuretics, monitoring of liver function, and interventions like paracentesis for ascites and oxygen therapy for respiratory support [14,15].

In Ethiopia, a liver disease of unknown origin was first identified in 2002 in the Tahtay Koraro district, specifically in the Kelakil village, located in the rugged, semi-arid, mountainous region of northwestern Tigray. The initial identification was suspected due to the ingestion of pyrrolizidine alkaloid-containing plants, such as *Ageratum conyzoids*. Later, in 2005, an outbreak of fatal liver disease was reported in the same district. By 2017, a study identified 179 possible cases of HVOD in the Tahtay Koraro district, with 83 deaths, resulting in a case-fatality rate of 46.3% [8,16]. These severe outcomes emphasize the urgent need to better understand and address this health crisis within the affected populations.

Initially, the condition was referred to as unknown liver disease (ULD). However, it was reclassified and named Hepatic Veno-occlusive disease (HVOD) following a comprehensive investigative study conducted by multidisplinary and multisectoral researchers [16]. This extensive study not only led to the renaming of the disease from ULD to HVOD but also identified its etiology, linking it to a toxin known as pyrrolizidine alkaloid (PA) derived from the plant *Ageratum conyzoides*. Despite this etiological clarity, persistent gaps in community understanding and disease control efforts continue, justifying further exploration of social and behavioral factors influencing disease management.

Despite the challenging circumstances in Tigray, including the collapse of healthcare infrastructure, health service delivery units [17], and low reporting rates for HVOD due to previous armed conflicts, a concerning trend has emerged. According to Tigray regional health bureau, there are high case reports from various districts in the region, and HVOD has also begun appearing in areas where it was previously unreported. This development raises skepticism among both health professionals and the community regarding its root cause. The skepticism is compounded by the association of HVOD with exposure to *Ageratum conyzoides*. A recent report from the regional health bureau indicates that HVOD is now affecting 16 districts within the region, resulting in a significant increase in both cases and fatalities. The combination of rising disease prevalence, lack of acceptance regarding its origins, and limited understanding of the disease’s etiology has heightened unease among local populations and authorities alike. This evolving epidemiology and community skepticism highlight the necessity of investigating perceptions and practices to inform culturally appropriate and sustainable interventions.

Given the existing challenges in HVOD-affected communities in northwestern Tigray, Ethiopia, conducting rigorous qualitative research is essential to comprehensively identify and analyze underlying issues, perceptions, and practices. This approach is particularly suited for understanding the “why” and “how” of complex health issues, providing valuable insights into the lived experiences of individuals and communities [18,19]. The objectives of this study include exploring sentiments regarding previous findings, examining community and healthcare professionals’ attitudes, practices, and acceptance of earlier HVOD interventions, and understanding community skepticism about HVOD’s root causes. These findings can help guide the development of appropriate measures and interventions.

## Methods

### Ethics statement

Ethical clearance was obtained from the Institutional Review Board (IRB) of the Tigray Health Research Institute (THRI) with a reference number (THRI-IRB/71/2025). Confidentiality of participants’ information was maintained. Permission from the Tigray Regional Health Bureau was obtained before commencing the study. At each selected site, local administrators were informed and consented to participate in advance. Verbal informed consent was obtained from all assessment participants. To address potential distress among participants affected by HVOD, trained data collectors were instructed to conduct interviews sensitively, and a referral mechanism was established with local health facilities to provide appropriate psychological or medical support if needed.

### Study area and period

The study was conducted in the Tigray Region of Northern Ethiopia from January to February 2025. Tigray is divided into seven administrative zones, comprising 93 districts (57 rural and 36 urban), and structured in 814 Tabias (753 rural and 61 urban). The region’s population is estimated to be more than seven million people, with the majority of inhabitants residing in rural areas. Before the severe war crisis, which was among the most devastating in recent regional history, the healthcare system included a workforce of over 25,000 professionals ranging from community health extension workers to specialists.

**Fig 1:**
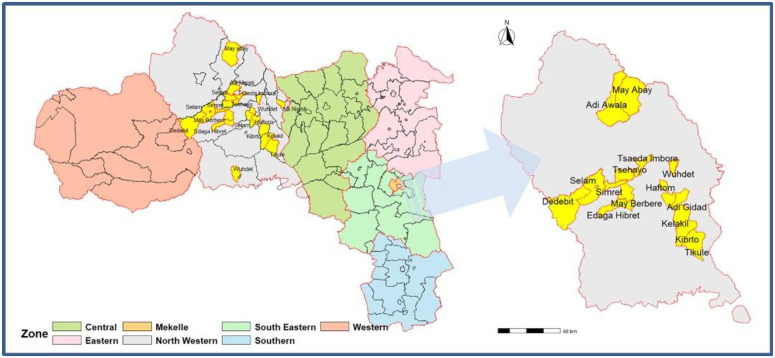
Map of the study areas in the northwestern zone of the Tigray Region, Northern Ethiopia. The base map was created using ArcGIS software. Administrative boundaries were obtained from the Humanitarian Data Exchange public domain dataset (https://data.humdata.org/dataset/cod-ab-eth), which provides shapefile formats. Spatial accuracy was validated through GPS field data collection.

The North Western Zone of Tigray was specifically targeted for this study due to its high burden of Hepatic veno-occlusive disease (HVOD). This zone borders Eritrea to the north, Amhara Region to the south, Central Zone to the east, and Western Zone to the west. It consists of fifteen districts: Asgede, Tsimbla, Laelay Adiabo, Seyemti Adiabo, Maekel Adiyabo, Laelay Koraro, Zana, Tahitay Adiabo, Tahtay Koraro, Laelay Tselemti,Tselemti May Tsebri Endabaguna Sheraro Shire Endasilassie (Fig 1). Public health services are provided through various facilities including health posts (108), health centers (38), primary hospitals (4), and general hospitals (2). Economically significant activities in these districts include smallholder mixed crop-livestock agriculture with crops like sorghum, maize, teff, finger millet faba bean pearl millet lentil sesame groundnut niger seed sunflower seed rice soybean wheat barley being commonly produced alongside livestock such as cattle, goats, sheep, donkeys, camels and chickens which are prominent features of the local economy. Additionally, traditional mining is widely practiced in the area.

### Study design

Both community and facility-based exploratory qualitative study was conducted to understand community sentiments regarding previous findings, which is crucial for assessing feelings, practices, and acceptance of earlier interventions related to HVOD. To facilitate dialogue, interview and discussion guides were utilized among selected groups and individuals from the study communities, district health offices and health facilities (S1 File).

#### At the community level.

In-Depth Interviews (IDIs) and Focus Group Discussions (FGDs) were conducted with community and religious leaders, victims of HVOD, and residents. Community and religious leaders provided valuable insights into local beliefs, cultural practices, and the social context surrounding health issues. Victims of HVOD shared their personal experiences, challenges, and perspectives on the disease. The IDIs allowed for an in-depth exploration of individual experiences, whereas the FGDs facilitated group discussions to gather collective opinions and perspectives.

#### At the facility level.

Key Informant Interviews (KIIs) were conducted with health professionals from health facilities and representatives from district health offices. Health professionals including, doctors, nurses, and other healthcare providers treating patients, offered expert insights into clinical practices, patient care, and health outcomes.

### Sample size and sampling technique

The districts of Asgede, Maekel Adiyabo, Zana, Tahtay Koraro, and Adi-Daero were purposively selected for this study due to their high burden of HVOD. From each district, one Tabia (the lowest administrative unit, typically comprising about 5,000 people) was randomly selected. Participants for focus group discussions (FGDs), key informant interviews (KIIs), and in-depth interviews (IDIs) were chosen using a purposive sampling strategy, aiming to select individuals with comprehensive knowledge relevant to the discussion topics. The participants in FGDs, IDIs and KIIs included victims and their families, community and religious leaders, healthcare leaders and health professionals. By employing a maximum variation purposive sampling technique, a diverse range of participants was identified to represent a broad spectrum of experiences and characteristics. The study included a total of three FGDs, sixteen IDIs, and seven KIIs. The sample size was determined based on the concept of theoretical saturation, defined as the point at which additional data collection no longer yields new insights relevant to the research questions. Data collection continued until no new themes or information emerged, ensuring that thematic saturation was achieved. This explicit confirmation of saturation enhances methodological rigor by demonstrating that the data collected sufficiently captured the range and depth of perspectives necessary for comprehensive analysis [20].

### Data collection procedure

Qualitative data were collected through In-Depth Interviews (IDIs), Focus Group Discussions (FGDs), and Key Informant Interviews (KIIs). The study included three FGDs with 8–10 participants each, sixteen IDIs and seven KIIs with health professionals, engaging community leaders, HVOD victims, healthcare leaders and professionals. Participants were selected based on their potential to provide relevant information about HVOD perceptions. FGDs and IDIs were conducted at local health posts and community centers, with assistance from health workers and Tabia leaders. Interview guides were developed in English (S1 File) and translated into Tigrigna, pre-tested, and revised. Daily debriefing session’s ensured new issues to be addressed. The FGDs lasted 50–80 minutes, while KIIs and IDIs lasted an average of 40 minutes. Data were recorded, translated into English, and reviewed by investigators.

### Data analysis

The qualitative data collected from focus group discussions (FGDs) and in-depth interviews (IDIs) with community representatives and key informant interviews (KIIs) were transcribed verbatim in the local language, Tigrigna, and then translated back into English by the investigator. The data were analyzed using a thematic analysis approach, facilitated by Atlas.ti software (version 7.5.4) for data management and coding. Both inductive and deductive coding methods were employed to ensure comprehensive analysis. Two investigators, Mekonnen Haileselassie, PhD (Public Health), and Mrs. Ziada Abdelhadi Abdelwahab, MA (Medical Sociology), who have authored several qualitative studies, independently coded the data to assess inter-coder reliability, resolving any discrepancies through discussion until consensus was reached. The codes were then reviewed for consistency and grouped according to the study objectives to ensure rigor and transparency in the analysis. Thematic analysis was applied to synthesize the main topics from categorized data, paying attention to variations and similarities in views. Themes were supported by direct quotes from participants, which were translated from Tigrigna into English.

## Results

### Socio demographic characteristics of participants

The study encompassed selected areas from each district within the Tigray region, highlighted in yellow on the zonal map. The inset provides a detailed view of these study areas, illustrating their spatial distribution and pinpointing the specific locations where data collection took place (Fig 1). The qualitative study included 42 participants (16 IDIs, 3 FGDs with 19 total participants, and 7 KIIs). The mean (±SD) age of the study participants was 45 (12.7) years. Over 80% of KII participants held a BSc degree or higher in their educational status. Males comprised 59.5% of the total study participants. Approximately 63% of community participants were illiterate. KIIs had work experience ranging from 6 to 32 years (Table 1).

**Table 1:** Socio-demographic characteristics of study participants in Tigray, Ethiopia.

Characteristics	IDI (N = 16)	KII (N = 7)	FGD (N = 3)
**Districts**			
Zana	8	3	13
T/koraro	5	2	6
Asgede	3	1	–
Endasilassie	–	1	–
**Age category in years**			
< 35	6	4	4
36-40	1	–	4
> 40	9	3	11
**Sex**			
Female	9	1	7
Male	7	6	12
**Level of education**			
No formal education	12	0	11
1–8^th^	3	0	6
9-12	1	0	2
Diploma	0	1	0
First degree	0	5	0
Second degree	0	1	0
**Working experience in years**			
6-15	–	5	–
> 30	–	2	–

### Community and health facility insights into HVOD with key themes and findings

Data from community- and facility-based in-depth interviews (IDIs), focus group discussions (FGDs), and key informant interviews (KIIs) were analyzed to explore perceptions and practices related to HVOD causes and prevention. Three key themes emerged: (1) current experiences, (2) barriers to HVOD management, and (3) participants’ suggestions (Table 2).

**Table 2:** Thematic framework of community perceptions and practices on hepatic veno-occlusive disease in Tigray, Ethiopia.

Themes	Categories	Codes
Current experiences	Knowledge and understanding	Awareness of HVOD origin; Understanding prevention methods; Knowledge of treatment options
	Unified support through inclusive leadership	Community leadership involvement; Collaborative decision-making; Support networks
Barriers to HVOD management	Community perspectives	Beliefs about HVOD causes; Skepticism about *Ageratum conyzoides* as cause; Local disease explanations
	Social barriers	Stigma associated with HVOD; Traditional beliefs; Fear and misinformation
	Systemic challenges	Political instability; Healthcare infrastructure gaps; Limited surveillance and reporting
	Economic constraints	Cost of healthcare; Access to medical services; Financial burden on affected families
	Intervention acceptance	Acceptance of scientific interventions; Trust in health authorities; Resistance to new methods
Participants’ suggestions	Healthcare provider training	Need for specialized HVOD training; Capacity building; Continuous education
	Community involvement	Engagement in awareness campaigns; Participation in intervention planning; Local knowledge integration
	Inclusion in national health programs	HVOD recognition in health policy; Resource allocation; Integration with existing health services

### Theme 1: Current experience

#### Knowledge and understanding.

The community’s understanding of HVOD is multifaceted, reflecting both a deep recognition of its severity and confusion about its nature. Participants widely acknowledged HVOD as a chronic and life-threatening condition with symptoms that progress from mild to severe. This understanding is summarized in the following quote:

*“There is no cure for it, and although it’s not transmittable between households, it is fatal. Once someone is infected, it eventually leads to death after prolonged suffering” (42 years, female, IDI).*

However, there is confusion regarding its transmissibility. Some participants questioned its non-transmissible status due to its prevalence in entire households:

*“Though it is considered non-transmissible, I doubt this, as it often affects entire households and even destroys many. However, seeing many unaffected households makes me confused about its transmission” (45 years, male, IDI).*

Moreover, the community highlighted significant gaps in healthcare providers’ ability to diagnose and manage HVOD effectively. Misidentification of the disease by healthcare providers was a recurring issue:

*“The disease remains undiagnosed for a long time, even when medical treatment is sought. Misidentification in its early stages, often mistaken for malaria or ‘Guaka,’ leads to delayed treatment” (50 years, female, IDI).*

*Healthcare professionals corroborated this knowledge gap, with one stating:*

*“When I was assigned to this district, I had no prior information about HVOD, and even senior colleagues did not inform me of its presence. I first became aware of it through the community” (27 years, male, KII).*

Furthermore, providers acknowledged the lack of proper training and protocols for HVOD diagnosis and treatment:

*“Currently, no training is provided for HVOD, and further training is required in HVOD diagnosis, case management, and treatment” (29 years, male, KII).*

#### Unified support through inclusive leadership.

Despite the community’s knowledge about HVOD, there is a significant lack of leadership support and coordinated efforts from healthcare providers, political leaders, and other stakeholders. Participants expressed frustration over the absence of structured interventions for awareness creation, prevention strategies, and disease-specific treatment:

*“Efforts to remove ‘Kewie Genene’ or Ageratum conzoides are interrupted mainly because the community and local leaders do not believe it is the cause. Currently, any government support, including free medication has stopped” (35 years, female, IDI).*

The community’s familiarity with HVOD symptoms contrasts with their disagreement about its cause. Many linked it to divine origins due to the lack of scientific understanding:

*“I have known this disease for decades—it weakens people slowly, leaving them without a cure. At first, even doctors mistook it for malaria, but later they called it ‘Himam Tselam Kebdi.’ Many believe it is from God, as its cause remains unknown” (70 years, male, IDI).*

Healthcare providers echoed concerns about systemic failures in addressing HVOD:

*“It is known about the current scenario because of the country’s failure... no one focuses on this movement for HVOD situations” (55 years, male, KII).*

Efforts to mechanically remove *Ageratum conyzoides*, previously suspected as a cause of HVOD, have largely been abandoned due to skepticism about its effectiveness:

*“The government once blamed the ‘Hagay Fetewe’ plant and forced us to clear it, but the disease persisted. If they were sure, they should have continued enforcing it until we saw results. Now, the issue is ignored, and people are left to suffer without proper medication. The real cause remains a mystery” (70 years, male, IDI).*

Participants also noted that resource constraints made large-scale removal impractical:

*“Decades ago, there was a campaign to remove the plant. But now how could farmers alone clear it after spending the entire summer struggling with other weeds?” (60 years, female, IDI).*

### Theme 2: Barriers to HVOD management

Managing hepatic veno-occlusive disease (HVOD) faces several barriers that complicate prevention, treatment, and control efforts. These barriers can be categorized into community perspectives, social barriers, systemic challenges, economic constraints, and intervention acceptance.

#### Community perspectives.

The Ageratum plant is ubiquitous in the community’s agricultural lands, homesteads, and un-ploughed areas. Despite its widespread presence, community members generally do not believe that the plant causes hepatic veno-occlusive disease (HVOD). They have used the plant for generations without issues, and animals that consume it do not get sick. As one participant noted,

*“Hagay Fetewe (Ageratum weed) had been part of our life for generations. Our mothers used it to mix with butter for hair treatment, believing it preserved the butter from melting. We also used it to sharpen traditional drinking utensils (Shkna)” (50 years, female, IDI).*

Another participant emphasized, *“I do not believe that Ageratum weed is the cause. It has been with us for a long time and is spreading rapidly. Animals also eat it, yet they do not suffer from the disease, even though scientific studies claim otherwise” (35 years, male, IDI).*

This skepticism is also shared by healthcare professionals, who find it challenging to convince communities of the scientific explanation. A KII participant from a healthcare facility stated, *“There is a significant difficulty since communities refuse to accept the scientific explanation of HVOD while simultaneously resisting the recommended remedies” (42 years, male, KII).*

Some community members attribute the disease to divine punishment or evil spirits. For example, *“The prophecy is stated like: first affect ‘Tsaeda Emba,’ spread across different communities at irregular intervals, and then fade away as it traveled east” (60 years, man, IDI).* Another participant mentioned, *“Initially, it was believed to be caused by the evil deeds of ‘Deftera’ (spiritual healers), but even after we left the area, the disease still persisted” (55 years, male, FGD).*

In contrast, some participants expressed moderate beliefs that the ageratum plant could be a potential cause of the disease. One key informant stated: *“The cause of HVOD could be the ageratum plant; however, the community is currently highly traumatized and fatigued from previous conflicts. Additionally, the plant has extensively spread over large areas, making it very difficult for the community to eradicate. Effective control may require technological interventions beyond community efforts” (34 years, male, KII).*

#### Social barriers.

Social stigma is a significant barrier to healthcare access for HVOD patients. Many fears being labeled as infected, which discourages them for early medical examinations. As one participant noted, *“People avoid early medical examinations, even when they recognize the symptoms, because they fear being labeled as infected. However, those who seek treatment early recover more effectively and survived” (30 years, male, FGD).*

This stigma also affects social interactions, as victims are excluded from community activities and struggle to work due to their condition. For instance, *“…it prevents people from engaging in hard labor. Victims feel weak and cannot work for their survival” (30 years, male, FGD).*

#### Systemic challenges.

The healthcare system faces several challenges in addressing HVOD. There is a lack of training for healthcare professionals on diagnosing and managing the disease, and no guidelines exist for treatment and prevention. A KII participant noted, *“Currently, there is a knowledge gap in diagnosing and managing HVOD cases, and the majority of the workforce is new” (42 years, male, KII).* Another participant stated, *“The main challenge is the lack of guidelines for HVOD management. Without proper guidelines, it is difficult to suspect HVOD cases” (34 years, male, KII).*

Some healthcare providers express skepticism regarding the effectiveness of available medical treatments for HVOD. As one key informant noted, *“We believe that medical interventions primarily serve to prevent complications and may only modestly prolong life, especially when patients present at advanced stages of the disease.” (30 years, Male, KII).* This perspective reflects broader concerns about the limitations of current therapeutic options for HVOD, compounded by delayed healthcare seeking behaviors that often result in diminished treatment efficacy.

The two-year war in Tigray has significantly damaged health facilities, and the government’s attention toward the treatment and prevention of hepatic veno-occlusive disease (HVOD) remains minimal. As one participant stated, “*There is no implementation or preventive strategy in place to deal with HVOD due to a lack of capability or favorable conditions” (35 years, male, KII).*

Furthermore, another participant noted, “*Government programs do not prioritize HVOD issues like they do for diseases such as HIV, TB, and malaria” (42 years, male, KII).*

#### Economic constraints.

Economic hardship is a major challenge for HVOD patients. Previously, the government provided free medication, but now patients must purchase it themselves, which is often unaffordable. This financial burden leads to delayed treatment and increased mortality. A participant highlighted:

*“A major challenge for victims is financial hardship. In the past, the government provided free medication, but now people must buy it themselves, which is a huge burden. Many die when they can no longer afford treatment” (35 years, female IDI).*

The community’s reliance on traditional remedies like holy water further complicates the situation. A participant highlighted, *“A major challenge for victims is financial hardship. In the past, the government provided free medication, but now people must buy it themselves, which is a huge burden. Many die when they can no longer afford treatment. Victims also first turn to religious remedies such as holy water or locally called Maychelot and ‘Tsebel” (35 years, female IDI).*

Some participants expressed the belief that no truly effective medicine exists for the disease. One participant stated, *“This condition is perceived as a consequence of our sins, and victims often seek remedies through holy water and traditional medicines rather than visiting health facilities.” (55 years, male, FGD).*

The cost of accessing healthcare, combined with the need to sell assets to cover expenses, exacerbates the economic strain on affected families. As one participant noted:

*“……now, four members of my family, including me, are completely dependent on medication. The difficulty in accessing it has worsened, forcing me to use my assets, including cattle, to cover the costs. However, it has become increasingly challenging as the medicine is now accessed privately, adding further worry to our misery” (60 years, male, IDI).*

#### Intervention acceptance.

Interventions aimed at controlling HVOD face significant resistance due to community skepticism about the cause of the disease. There is a lack of proven referral linkages and no incorporation of HVOD into existing health programs. A KII participant emphasized:

*“It is necessary for women development army and village health leader to define community cases and structure them so they could be referred easily, however, there is no such practice in the ground” (29 years, male, KII).*

Effective interventions require strong stakeholder engagement, clear implementation strategies, and addressing potential barriers within the target population. However, without political will and community engagement, these efforts are likely to face significant challenges. As participants noted, *“Currently, the tabia leaders do not give attention to HVOD. Formerly, they had awareness, but with the current situation, these activities are not performed at all” (31 years, male, KII).*

Additionally, the community’s past experiences with interventions have been unsuccessful, further complicating acceptance. For example, *“At first, when the disease was identified, we said that it was caused by contaminated drinking water by the plant. As a result, we were instructed to bury our water sources but failed to bring solution” (50 years, female, IDI).*

Another participant mentioned, *“We followed the authorities’ instructions and covered contaminated water sources. However, the disease still exists in other areas” (50 years, male, FGD).*

*“The government once blamed the ‘Hagay Fetewe’ plant and forced us to clear it, but the disease persisted. If they were sure, they should have continued enforcing it until we saw results. Now, the issue is ignored, and people are left to suffer without proper medication. The real cause remains a mystery” (70 years, male, IDI).*

Before the conflict, the disease received significant attention from both the health and agriculture sectors. The government raised awareness among communities on how to clear the weed Ageratum, which contributed to controlling the disease, and medical support was provided at health facilities. As one participant stated, *“Before the conflict, relatively the government had attention to the disease by creating awareness among the communities how to clear the plant Ageratum, and there was medical support for the victims at the health facilities.” However, now the disease is ignored by the government” (45 years, male, FGD).*

### Theme 3: Participants’ suggestions

#### Healthcare provider training.

Participants highlighted the need for targeted training programs to improve healthcare providers’ capacity to manage Hepatic Vein Occlusive Disease (HVOD). Standardized guidelines should be developed and integrated into training sessions to enhance skills in diagnosing, treating, and preventing HVOD. As one participant stated:

*“Continuous professional development for healthcare workers is crucial for addressing gaps in knowledge and ensuring early detection and effective management of cases” (55 years, female, KII).*

One key informant interviewee also stated, *“The availability of laboratory services, drugs, and supplies for HVOD cases is very limited; thus, equipping health facilities with the necessary equipment, drugs, and trained personnel is crucial to improving their readiness.” (42 years, female, KII).*

#### Community involvement.

Community engagement was identified as a critical component in combating HVOD. Participants recommended raising awareness through health campaigns and mobilizing local leaders, such as health extension workers, to encourage health-seeking behaviors. Sustained efforts in community sensitization were emphasized as essential for prevention and early intervention. As noted,

*“Better awareness levels can be achieved through health awareness campaigns in the community” (55 years, female, KII).*

Participants claim that additional resources, in addition to community mobilization, are needed because the coverage of ageratum is vast. As one participant stated*:*

*“Decades ago, there was a campaign to remove the plant, but now how could farmers alone clear, after spending the entire summer struggling with other weeds?” (60 years, female, IDI)*

#### Inclusion in national health programs.

Participants called for the inclusion of HVOD in national health programs alongside diseases such as HIV, TB, and malaria. Strengthening active surveillance systems such as case investigations and line-listing was seen as vital for improving disease monitoring and response.

*“Inclusion of HVOD in national health programs such as those addressing HIV, TB, and malaria; strengthening active surveillance, including case investigations and line-listing; and ensuring that WHO and other non-governmental organizations (NGOs) concerned with community health focus on HVOD are critical priorities.” (42 years, male, KII).*

“Multi-sectoral collaboration was strongly recommended to establish robust surveillance mechanisms and secure dedicated resources for HVOD management, including the provision of medications and preventive materials. Sustained support for relocated residents affected by HVOD was also identified as a critical priority. One participant emphasized the importance of inclusive efforts, stating,

*“All sectors, including agriculture, education, water, energy, and non-governmental organizations (NGOs) concerned with community health, should actively participate in HVOD prevention and eradication” (42 years, male, KII).*

## Discussion

The purpose of this study was to explore the perceptions and experiences of the community and healthcare professionals regarding HVOD management in northwestern Tigray, Ethiopia. The findings aim to provide critical insights for developing effective intervention strategies to improve HVOD prevention, management, and overall patient outcomes among affected communities and healthcare workers.

This study indicated that the majority of FGD, IDI, and KII participants explained that their communities reject the scientific explanation of Ageratum as the cause of HVOD, instead attributing it to divine punishment or other supernatural beliefs. Cultural beliefs, values, and rituals have an impact on how people enter the health care system and how they care for themselves. While some cultural practices are acceptable, others may be detrimental to one’s health. This study is similar with the study conducted in Bamar the misperception about the cause of the disease, reveals several cultural behaviors associated with diabetes [21]. The possible cause might be despite previous efforts to educate the community, one major issue is the community’s carelessness regarding the cause of HVOD, partly due to a lack of health education and there are no regular intervention programs to understand the scientific origin of the condition, and there is inadequate training for healthcare workers to explain the reason of the disease in depth.

This misconception of the cause of the disease may render the general public to be less practical in taking measures to prevent HVOD. Misconceptions concerning HVOD have impacts on their dietary intake, prevention activity, adherence to medical management and intervention measures. The reason for dominant misconceptions about HVOD is multi-factorial. These include deficiency of knowledge about HVOD, poor education and cultural beliefs. It is very vital to identify the main misconceptions in the community to be able to inauguration proper health education programs for monitoring, prevention and implementation of effective intervention measures.

The systemic challenges prevent the proper provision of the services for HVOD early diagnosis and treatment activities, and some community members reported that healthcare providers frequently misdiagnose the disorder, leading to poor management. Armed conflicts have significant and complex effects on healthcare systems. It disrupts vital services, increase vulnerability, and negatively impact the health and well-being of affected populations. In Ethiopia’s Tigray region war conflict affected the region for more than two years.

The healthcare delivery and infrastructure have been severely impacted, resulting in a complicated and worsening public health crisis including HVOD early prevention diagnosis, management and treatment. The loss of healthcare infrastructure, including hospitals, clinics, and supply chain has severely affected [22]. This finding is consistent with countries whose health systems disintegrated during wartime [23–25].

The lack of community engagement affected the effectiveness of the intervention and the effective intervention for HVOD prevention like mechanical removal of the ageratum plant according the study conducted in Tahtay koraro district in Tigray [16]. The effectiveness of community engagement and professionals drove health-promoting interventions and behaviors, with little or no input from the target communities [26]. Community engagement refers to including communities in decision-making, planning, design, and governance processes [27]. Studies indicate that community engagement can lead to positive social outcomes [28]. The communities in the HVOD-affected areas are currently not executing effective interventions by engaging the communities, and the impact of the military conflict is affecting the framework that coordinates the community’s active participation in intervention programs.

In order to effectively reduce stigmatization and discrimination progress the experience of HVOD service users, anti-stigma interventions essential to be intended, attuned and implemented in exclusive local context. The stigma and discrimination of the area and individuals afflicted by HVOD can lead to discrimination and social isolation in their marriage relationships, worsening the problems and deteriorating the individuals’ physical and psychological well-being [29,30].

The relationship between human health, biodiversity, and land-use changes is complex and varies across time and space, making it challenging to address public health issues effectively [31]. To enhance early detection, prevention, and response to public health threats, multisectoral collaboration is essential at all levels [32]. However, this study revealed that in HVOD-affected areas, there is a lack of coordinated and consistent preventive initiatives. Multisectoral collaboration in these areas was poor, with limited involvement of all relevant sectors in the prevention and control of the disease.

Clear and accessible guidelines at health facilities are essential for the effective management of diseases, as they provide structured approaches for prevention, diagnosis, and treatment. The absence of such guidelines can significantly hinder disease control efforts [33,34]. However, in this study, most of the key informant participants reported that the catchment health facilities lack guidelines for awareness creation, prevention, and treatment of HVOD. Existing guidelines primarily focus on the pathological characteristics of the disease, but most facilities affected by HVOD do not have comprehensive guidelines to prevent, diagnose, manage, and treat the disease.

Although political leaders play a critical role in mobilizing communities and legitimizing public health campaigns [35], in this study, political leaders show little attention to HVOD, and community mobilization efforts against the disease are significantly hampered. Similarly, effective health system governance, which involves collective decision-making and coordination across various stakeholders, is crucial for successful disease management.

### Strengths and limitations

The strengths of this study include the collection of high-quality data by an experienced research team with extensive expertise in qualitative methods. The inclusion of diverse participant groups, such as community and religious leaders and healthcare professionals, provided rich insights through Key Informant Interviews (KII), In-Depth Interviews (IDI), and Focus Group Discussions (FGDs). Throughout the research, the team engaged in ongoing reflexivity, critically examining how their positionalities, academic backgrounds, and linguistic competencies could influence data collection, analysis, and interpretation. Regular reflective discussions helped identify and mitigate potential biases and power dynamics, thereby enhancing transparency, reducing bias, and strengthening the study’s methodological rigor and credibility. However, limitations include the potential lack of representativeness due to purposive sampling and possible loss of nuances during translation from Tigrigna to English. To mitigate this, interviews were audio-recorded, transcribed word-for-word in Tigrigna, and verified during translation. Another limitation of this study is the transferability of its findings, as it was conducted in a post-conflict setting. The unique social, economic, and health system challenges in this context may influence community perceptions and service delivery differently than in stable settings. Therefore, caution is advised when generalizing these results beyond similar post-conflict or resource-limited areas. Thematic saturation is typically assessed retrospectively after data coding, which can bias saturation determination. In this study, daily debriefing sessions were conducted during data collection to incorporate emerging issues into subsequent interviews, allowing a more dynamic and prospective saturation assessment. Despite these limitations, the study provides valuable insights into community perceptions and practices regarding HVOD, which are critical for informing effective interventions.

## Conclusions

Hepatic veno-occlusive disease (HVOD) has become a critical public health issue in Ethiopia, particularly in the Tigray region, where cases and fatalities have been rising. The disease may link with pyrrolizidine alkaloid (PA) exposure, primarily from the plant *Ageratum conyzoides*, although skepticism regarding this cause persists within affected communities. Many individuals attribute HVOD to supernatural causes or divine punishment, which has fostered stigma and delayed healthcare-seeking behavior. This stigma, coupled with a lack of awareness, exacerbates the disease’s impact by preventing timely diagnosis and treatment. Furthermore, healthcare system challenges, including insufficient training of healthcare providers, frequent misdiagnoses, limited treatment options, and financial barriers, have significantly worsened patient outcomes.

Institutional and structural barriers further complicate HVOD management. The disease has not been prioritized in national health programs, resulting in inadequate surveillance and intervention strategies. Armed conflict in the region has strained health resources, damaged facilities, and disrupted logistical systems necessary for effective disease management. Additionally, community-based interventions such as mechanical removal of *Ageratum conyzoides* have been inconsistently implemented due to resource limitations and a lack of trust in their effectiveness. Addressing these challenges requires coordinated efforts to strengthen healthcare infrastructure, integrate HVOD into national health priorities, and implement evidence-based interventions effectively. Central to this approach is the incorporation of community engagement strategies in upcoming interventions, as active involvement of affected communities enhances disease understanding, fosters ownership, and promotes sustainable control. To support these efforts, we include proposed content for provider training alongside practical community engagement strategies to better guide policymakers and program planners.

## Supporting information

S1 File Interview Guides for (A) Focus Group Discussion(B) Individual Interview, and (C) Guide for key informants. (DOCX)
