# Supplementary material for: Community perceptions and practices on hepatic veno-occlusive disease in Tigray, Ethiopia: An explorative study challenging the attribution to Ageratum conyzoides
Source: PLoS Negl Trop Dis. 2025 Oct 15;19(10):e0013621. doi: 10.1371/journal.pntd.0013621 (PMC12543276; doi:10.1371/journal.pntd.0013621)
Supplement: S1 File — (DOCX) [file pntd.0013621.s001.docx]

**S1 File: Table 1 Guiding questions for qualitative data collection on HVOD: FGD, IDI, and KII participants**

| Participant Category | Key Questions |
| --- | --- |
| FGD (Community members, patients) | - What do you know about HVOD and its causes?  - How is HVOD viewed in your community?  - What prevention and treatment methods do you know?  - How are community leaders involved?  - What support is available for affected people?  - What beliefs or stigma affect HVOD management?  - What challenges exist in accessing care?  - How do you feel about scientific interventions?  - How can healthcare providers improve?  - How can the community be involved more?  - Should HVOD be part of national health programs? |
| IDI (Healthcare providers, health workers) | - What is your understanding of HVOD?  - What prevention and treatment do you provide?  - How do you involve community leaders?  - What beliefs or social barriers affect care?  - What systemic challenges do you face?  - How do economic factors affect patients?  - How do patients accept scientific interventions?  - What training do you need?  - How can community involvement improve?  - Should HVOD be integrated into health programs? |
| KII (Leaders, policymakers, officials) | - How aware are you of HVOD and its impact?  - What is the role of leadership in HVOD management?  - How are support networks organized?  - What community beliefs affect policy?  - How do stigma and misinformation impact efforts?  - What systemic and economic challenges exist?  - How accepted are scientific interventions?  - What policies support healthcare training?  - How can community involvement be enhanced?  - What steps are taken to include HVOD in health programs? |
